# Whole genome sequencing of *Streptomyces actuosus* ISP-5337, *Streptomyces sioyaensis* B-5408, and *Actinospica acidiphila* B-2296 reveals secondary metabolomes with antibiotic potential

**DOI:** 10.1016/j.btre.2021.e00596

**Published:** 2021-02-09

**Authors:** Haley M. Majer, Rachel L. Ehrlich, Azad Ahmed, Joshua P. Earl, Garth D. Ehrlich, Joris Beld

**Affiliations:** Department of Microbiology and Immunology, Drexel University College of Medicine, 245 N 15^th^ St, Philadelphia, PA 19102, USA

**Keywords:** Whole genome sequencing, AntiSMASH, Thiopeptides, Mass spectrometry

## Abstract

*Streptomycetes* are bacteria of biotechnological importance since they are avid producers of secondary metabolites, including antibiotics. Progress in genome mining has recently shown that *Streptomyces* species encode for many biosynthetic gene clusters which are mostly unexplored. Here, we selected three Actinomycetes species for whole genome sequencing that are known to produce potent thiopeptide antibiotics. *Streptomyces actuosus* biosynthesizes nosiheptide, *Streptomyces sioyaensis* produces siomycin, and *Actinospica acidiphila* is a member of the *Actinomycete* subfamily. Bioinformatic analyses demonstrated diverse secondary metabolomes with multiple antibiotic-encoding gene clusters. Detailed mass spectrometry analysis of metabolite extracts verified the active expression of nosiheptide and siomycin from *S. actuosus* and *S. sioyaensis* while fractionation of the bacterial extracts and subsequent challenge against *Staphylococcus aureus* demonstrated potent antibiotic activity of fractions containing these compounds. Whole genome sequencing of these species facilitates future bioengineering efforts for thiopeptides and characterization of relevant secondary metabolites.

## Highlights

• Whole genome sequencing of Actinomycetes reveals metabolic potential.
• High quality genomes are necessary for mining of biosynthetic gene clusters.
• Characterization of thiopeptides by high resolution mass spectrometry.
• Thiopeptides are potent antibacterials against *Staphylococcus aureus*.

## 1 Introduction

The multi-faceted problem of microbial resistance to antibiotics combined with human behavior has led to the antimicrobial resistance crisis [1]. In 2015, already declared a global health emergency [2], the WHO reports 700,000 deaths each year of multidrug resistant organisms with a predicted 10 million deaths in 2050 [3]. In the US, more than 35,000 deaths caused by antibiotic-resistant infections are reported in the latest CDC report [4]. Although a crisis with many underlying causes, an important factor is the “void” in antibiotic development since the late 1980s. After WHO and CDC crisis reports in 2015, more than 40 antibiotics have entered the drug approval pipeline [5] but 75 % are re-formulations of old antibiotics. Although effective in the short term, it is crucial to discover new targets and antibiotics with novel scaffolds to combat antimicrobial resistance in the long-term.

More than 75 % of antibiotics are derived from natural products [6,7]. Natural products are small- to medium-sized molecules produced by secondary metabolism in all kingdoms of life. Most natural products with antibiotic activity have been isolated from plants, fungi, and bacteria [7,8]. These molecules were discovered through a classical natural product discovery pipeline in which large amounts of biomass are extracted, the metabolites fractionated, and characterized [9]. This pipeline has several disadvantages, which partially resulted in closure of many pharmaceutical industry natural product divisions. A major disadvantage is that in 99.99 % of the cases, the same molecules that were previously discovered are found [10]. Dereplication, recognition and elimination of known molecules early in the screening process, is therefore a key process in natural product discovery.

In the past ten years, a renaissance has occurred in natural product discovery, driven by knowledge of biosynthesis, bioinformatics, and whole genome sequencing [8,11]. Since the early 1990s, we know that the natural product families of polyketides and non-ribosomal peptides are biosynthesized by biosynthetic gene clusters (BGCs). BGCs are operons of genes, from which the encoded proteins are responsible for the metabolism (including production, transport, resistance, and regulation of expression) of a natural product. Polyketide synthases and non-ribosomal peptide synthetases have a modular and often repetitive architecture, which makes it possible to mine genomes for their BGCs [[12], [13], [14]]. The antiSMASH server is a culmination of BGC mining algorithms, facilitating an easy way to predict what natural products an organism can make based on their genome sequence [15]. The modern natural product discovery pipeline thus starts with whole genome sequencing, followed by computational mining for BGCs, selection of unique BGCs, and subsequent cloning of the selected BGCs in an easy-production host. This pipeline, using modern computational and microbiology techniques, eliminates the need for dereplication, streamlining natural product discovery.

Whole genome sequencing (WGS) is an absolute requirement for the modern natural product discovery pipeline [8,9,16]. Many natural products have been found in *Streptomyces* species and both Illumina and PacBio sequencing have been used for WGS of these bacteria. Whereas Illumina is economical and gives short reads, PacBio is relatively expensive but provides long reads. Long reads enable the assembly of high-quality genomes with few contigs and bypasses the inaccuracies generated by the high G + C content of *Streptomyces’* genomes [17,18]. High quality genomes are essential for accurate computational mining for BGCs. Sequencing *Streptomyces clavuligerus* using Illumina and PacBio sequencing showed that 30 % of annotations were wrong and inaccurate nucleotides were found when using Illumina sequencing [19,20]. Illumina sequencing is however by far the favorite microbial genomics sequencing platform, but especially for the purpose of BGC analysis, PacBio sequencing is attractive. In contrast to Illumina sequencing, PacBio sequencing reads can span entire BGCs, which range in size from 1 to 200 kb. This is important for discovery of intact BGCs including transport, resistance, and regulatory genes. Computational prediction and future bioengineering studies rely on accurate and full-length sequences of BGCs.

*Streptomyces* are a major source of natural products, including antibiotics like chloramphenicol and streptomycin [21]. They are soil bacteria with relatively large genomes that have been shown to encode 8–83 secondary metabolite BGCs per genome [22]. However, this number may be overstated since many *Streptomyces* genomes are highly fragmented which can result in predictive tools misidentifying “new” clusters. Since many of these bacteria were isolated decades ago, the natural products they produce under laboratory conditions are often known, but their genomes have often never been sequenced. Here, we are comparing the previously sequenced *Streptomyces actuosus* NRRL ISP-5337^T^, known to produce nosiheptide [23], derived from the ATCC collection [24] to that of *Streptomyces actuosus* NRRL ISP-5337^T^ derived from the USDA ARS collection. In parallel, we sequenced *Streptomyces sioyaensis* NRRL B-5408^T^, known to produce siomycin, as the previously published *Streptomyces sioyaensis* NRRL B-5408^T^ genome scaffold is highly fragmented and difficult to analyze [25,26]. In addition, we sequenced *Actinospica acidiphila* B-2296, a newly identified *Actinospica acidiphila* strain that has a high level of genome relatedness to *Streptomyces* species [27]. Nosiheptide, siomycin, and thiostrepton are thiopeptides, large macrocyclic peptides that are extensively post-translationally modified [[28], [29], [30], [31]]. Thiopeptides have been shown to have many functions beneficial to human health, including antimalarial [32], anticancer [33], and immunosuppressive activities [34]. Importantly, thiopeptides are potent antibiotics with unique modes of action but not used in the clinic due to insolubility [35,36].

We sequenced these three genomes using PacBio RSII circular consensus sequencing which generated between 7 and 21 contigs per genome. Bioinformatic analyses of the genomes showcases the biosynthetic potential of these Actinomycetes species. We also verified, by liquid chromatography mass spectrometry, the production of thiopeptides, partially purified the natural products, and showed potent antibacterial activity against *Staphylococcus aureus*. WGS of these three Actinomycetes species enables future heterologous expression and bioengineering of BGCs of these bacteria. Exploration of the biosynthetic potential of these species coupled to bioengineering advances the field of natural product discovery and expands the reservoir of new antibiotics to address the antimicrobial resistance crisis.

## 2 Materials and methods

### 2.1 DNA sequencing

*Streptomyces actuosus* ISP-5337^T^, *Streptomyces sioyaensis* B-5408^T^, and *Actinospica acidiphila* B-2296 (abbreviated throughout as *S. actuosus*, *S. sioyaensis*, *and A. acidiphila*) were obtained from USDA/NRRL as lyophilized cells which were resuspended in 5 mL of Modified Bennett’s Medium (BEM broth) [37] and grown at 30 °C at 220 rpm for 5–7 days. High-quality bacterial genomic DNA was extracted following the salting-out method as previously described [38]. The gDNA preparations were pooled and stored at −20 °C in 2 mL of TE buffer. Bacterial identity was verified by amplifying the 16S rRNA sequences by PCR using forward primer 27F and reverse primer 1492R and Sanger sequencing. The 16S rRNA sequences were compared with the deposited 16S sequences at NCBI using BLAST. The quantity and quality of extracted gDNA was determined spectrophotometrically. DNA was processed according to the guidelines provided by PacBio for 10 kb template preparation. Each genome was sequenced on the PacBio RS II with one SMRTcell per genome. After sequencing, each genome was assembled using the PacBio Hierarchical Genome Assembly Process version 2 (HGAP_Assembly.2) [39], contigs with poor coverage or QV scores were removed with a custom Python script, contigs were merged using Circlator (v 1.0.2), and polished using PacBio’s resequencing pipeline. The resulting assemblies were annotated using Prokka v1.11 [40] with a custom genus database [41]. The genomes are uploaded to NCBI as S_actuosus_ISP-5337 (JABZEN000000000), S_sioyaensis_B-5408 (JABZEL000000000), and A_acidiphila_B-2296 (JABZEM000000000) under BioProject ID PRJNA632577.

### 2.2 Protein family identification

Cluster of Orthologous Groups analysis predicted the total number of protein families across the sequenced genomes by first scanning the Prokka-annotated amino acid files using InterProScan [42]. The results from the InterProScan analysis were submitted to EggNOG [43] for annotation and classification of protein families. The annotated protein families were plotted based on the number of total predicted proteins per category.

### 2.3 Secondary metabolite biosynthetic gene cluster prediction

The Prokka-annotated whole genome nucleotide files were mined for the presence of secondary metabolite biosynthetic gene clusters (BGCs) using antiSMASH 5.0 and features KnownClusterBlast, ActiveSiteFinder, ClusterBlast, Cluster PFam analysis, and SubClusterBlast [15,44].

### 2.4 Phylogenetic analysis

The 16S rRNA sequences of *S. actuosus*, *S. sioyaensis*, and *A. acidiphila* were aligned with all available *Streptomyces* full-length 16S rRNA sequences stored in the NCBI refseq database using the Mafft alignment version 7 sub-program Einsi [45,46] for high quality alignments. The 16S rRNA sequence alignment was visualized to verify the integrity of the alignment using Java Alignment Viewer [47]. A phylogenetic tree was generated from the alignment file using FastTree version 2 with maximum likelihood nearest-neighbor interchanges and minimum-evolution subtree-pruning-regrafting [48]. The tree was analyzed using FigTree [49] with midpoint rooting.

### 2.5 Whole genome relatedness

The average nucleotide identity (ANI) of the assembled nucleotide files of *S. actuosus*, *S. sioyaensis*, and *A. acidiphila* was calculated against the whole genome sequences of the strains used for 16S rRNA sequence analysis using PYANI 0.2.10 method MUMmer [50]. This method calculates nucleotide identity by pairwise sequence alignment which results in an overall average similarity of the genomes independent of sequence length.

### 2.6 Secondary metabolite extraction and analysis

A single colony of *S. actuosus*, *S. sioyaensis*, and *A. acidiphila* was inoculated in 5 mL of BEM broth [37] in duplicate and grown at 30 °C at 220 rpm for 3 days. The 5 mL cultures were then transferred to 500 mL of BEM broth in duplicate and grown for 5–7 days at 30 °C and 220 rpm. *S. sioyaensis* is known to produce thiopeptide siomycin which can be extracted from the bacterial cells using an organic solvent [25]. *S. actuosus* is known to produce nosiheptide which can also be extracted using an organic solvent [23,51]. Here, approximately 28.5 ng/mL and 11.4 ng/mL of siomycin and nosiheptide were produced in BEM broth, respectively. *A. acidiphila* bacterial cells were extracted using the same experimental setup as *S. sioyaensis* and *S. actuosus*. 500 mL broth cultures of each strain were harvested by centrifugation at 7000 rpm and 6 °C for 15 min. Metabolites were extracted from the cell pellet by incubating each pellet in 25 mL ethyl acetate (EtOAc) with rigorous shaking at room temperature overnight. The duplicate EtOAc phases were pooled and evaporated overnight. Crude extract (4 mg, 9 mg, and 8 mg, resp.) from *S. actuosus*, *S. sioyaensis*, and *A. acidiphila* was recovered.

### 2.7 Flash chromatography

The crude extracts from each strain were dissolved in 3 mL of EtOAc and loaded on 100 g of silica gel equilibrated in hexanes. Compounds were eluted with 100 mL of each eluents: hexanes, 1:1 hexanes:EtOAc, EtOAc, 1:1 EtOAc:methanol, and methanol. The fractions were collected in 50 mL increments and evaporated overnight. Each fraction was resuspended in 1 mL of methanol and stored at −20 °C.

### 2.8 Mass spectrometry of extracts

The metabolites in the crude extracts as well as the fractionated extracts were analyzed on a Waters Acquity I-Class UPLC system coupled to an Acquity TUV detector and Synapt G2Si HDMS mass spectrometer in positive ion mode with a heated electrospray ionization (ESI) source in a Z-spray configuration. LC separation was performed on a Waters Acquity UPLC BEH 1.7 μm 2.1 × 50 mm column using an 0.6 mL/min gradient of 95/5–15/85 A/B in 4 min followed by washing and reconditioning the column. Eluent A is 0.1 % formic acid in water and B is 0.1 % formic acid in ACN. Conditions on the mass spectrometer were as follows: capillary voltage 0.5 kV, sampling cone 40 V, source offset 80 V, source 120 °C, desolvation 250 °C, cone gas 0 L/h, desolvation gas 1000 L/h and nebulizer 6.5 bar. The analyzer was operated in resolution mode and low energy data was collected between 100 and 2000 Da at 0.2 s scan time. MS^e^ data was collected using a ramp trap collision energy 20−40 V, and masses were extracted from the TOF MS TICs using an abs width of 0.005 Da.

### 2.9 Antibacterial activity assays

*Staphylococcus aureus* ATCC 29213 and *Escherichia coli* DH5α were challenged with the metabolite fractions from *S. actuosus*, *S. sioyaensis*, and *A. acidiphila* to observe antibiotic activity per fraction. *S. aureus* or *E. coli* frozen glycerol stock were inoculated into 5 mL of Luria Bertani (LB) broth and grown at 37 °C and 220 rpm for 16−18 h to an OD600 of 2. The overnight cultures were diluted 1000-fold in 5 mL of LB broth. Six dilution cultures were prepared and incubated at 37 °C and 220 rpm for 80 min to a final OD600 of 0.01. We challenged 95 μL of 10^6^ CFU/mL of *S. aureus* to 5 μL of each culture extract fraction in duplicate then measured the antibiotic activity of the fractions by growth curve analysis using TECAN plate reader Infinite 200 M PRO. The OD600 was measured by averaging 4 reads per well per time point. The readings were taken every 15 min while the plate was maintained at 37 °C with shaking. The duplicate challenges were averaged for each time point and normalized to the methanol solvent control. The antibacterial activity assay was repeated 3 times for each strain and the standard deviation was calculated for each time point.

## 3 Results

### 3.1 Whole genome sequencing

*De novo* genome assembly was completed for *Streptomyces sioyaensis* B-5408, *Actinospica acidiphila* B-2296, and *Streptomyces actuosus* ISP-5337 using PacBio RSII technology and assemblies were annotated using Prokka [41] (Table 1). Each organism sequenced in this report was obtained from the United States Department of Agriculture (USDA) ARS culture collection (NRRL) as type strains. The species ordered were *Streptomyces cyaneus* B-2296^T^, *Streptomyces actuosus* ISP-5337^T^, and *Streptomyces sioyaensis* B-5408^T^. After sequencing, taxonomy analysis using GDTBD-Tk showed a high similarity of *S. cyaneus* B-2296^T^ to *Actinospica acidiphila* (∼95 %) [52]; therefore, the *S. cyaneus* B-2296^T^ isolate reported in this study is published as *Actinospica acidiphila* B-2296.

**Table 1:** Whole genome sequencing results summary for *Streptomyces actuosus* ISP-5337, *Streptomyces sioyaensis* B-5408, and *Actinospica acidiphila* B-2296. *Streptomyces and *Actinospica* genomes have been shown to be linear [27,53,80].

Strain	Genome		%GC	Number of Contigs
*Streptomyces actuosus* ISP-5337	Linear*	8,174,149 bp	72.5047	7
*Streptomyces sioyaensis* B-5408	Linear*	7,862,213 bp	71.5601	21
*Actinospica acidiphila* B-2296	Linear*	7,519,806 bp	72.4436	7

**Fig. 1:**
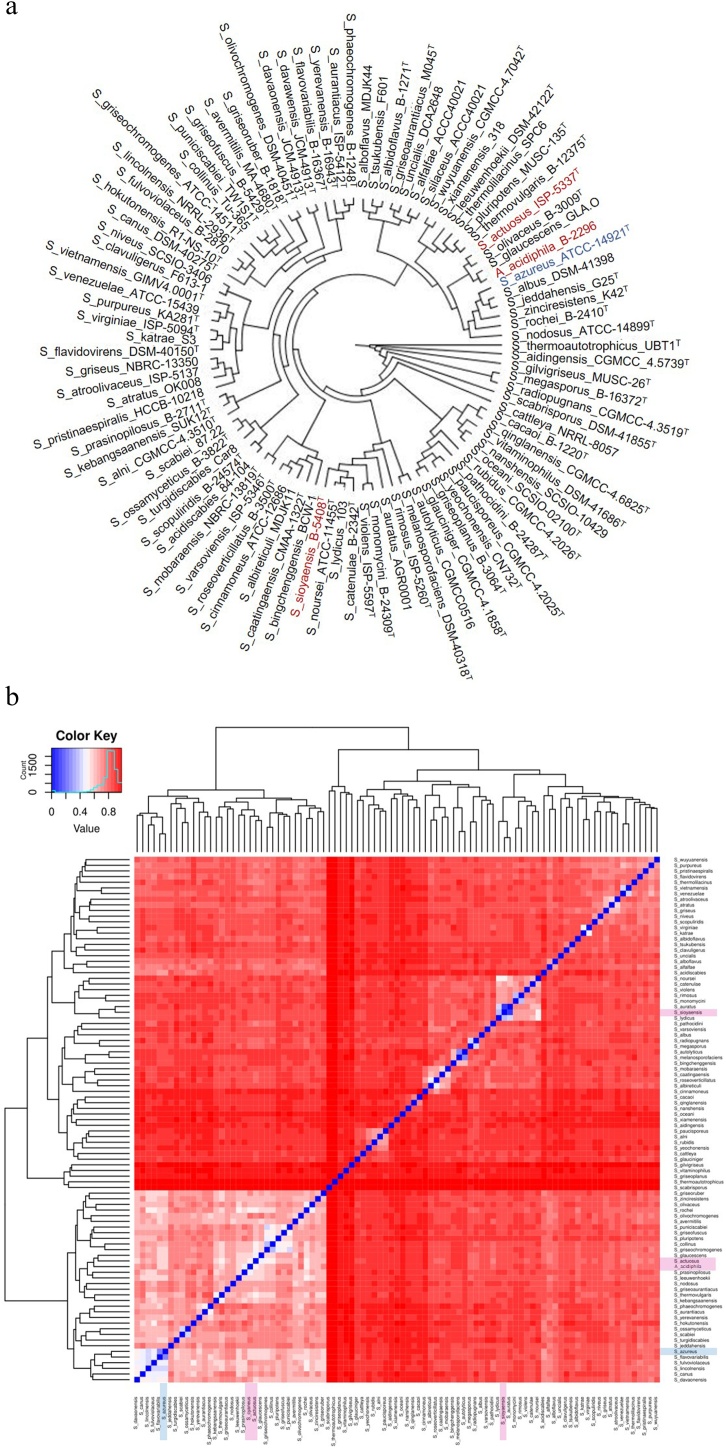
*Streptomyces* species relatedness. a) The 16S rRNA sequences of *S. actuosus* ISP-5337^T^, *S. sioyaensis* B-5408^T^, and *A. acidiphila* B-2296 were aligned with all full-length 16S rRNA sequences stored in the NCBI refseq database using the Mafft alignment version 7 [45] sub-program Einsi [46] for high quality alignments. The 16S rRNA sequence alignment was visualized to verify the integrity of the alignment using Java Alignment Viewer [47]. A phylogenetic tree was generated from the alignment file using FastTree version 2 with maximum likelihood nearest-neighbor interchanges and minimum-evolution subtree-pruning-regrafting [48]. The tree was analyzed using FigTree [49] with midpoint rooting. b) The average nucleotide identity (ANI) of *S. actuosus* ISP-5337, *S. sioyaensis* B-5408, and *A. acidiphila* B-2296 was calculated against the whole genome sequences of *Streptomyces* species in the NCBI refseq databased using PYANI 0.2.10 method MUMmer [50].

*Actinospica acidiphila* is a relatively newly identified Actinomycete sub-family member [27,53] that our data show to be closely related to some *Streptomyces* species (Fig. 1). The genome of *Actinospica acidiphila* was aligned to the reference genome of *A. acidiphila* B-24431 and resulted in 94 % similarity (Fig. S5). Importantly, the reference sequence of *A. acidiphila* B-24431 is reported as a fragmented scaffold that is difficult to analyze bioinformatically. The *A. acidiphila* B-2296 genome presented in this report is comprised of 7 contigs and a complete genome scaffold. The genome of *Streptomyces actuosus* ISP-5337 was aligned with the previously published type strain *Streptomyces actuosus* ISP-5337^T^ and resulted in 97 % similarity (Fig. S6), as well as the genome of *Streptomyces sioyaensis* B-5408 was aligned to the recently published *Streptomyces sioyaensis* DSM 40032 which resulted in 95 % similarity (Fig. S7).

Assembly resulted in linear genomes for each organism and an average GC content of approximately 72 %. Importantly, *S. actuosus* ISP-5337 and *A. acidiphila* B-2296 resulted in 7 contigs while *S. sioyaensis* B-5408 resulted in 21 contigs. This many contigs are unusual for PacBio sequencing; however, our Blue Pippin size selection parameters were selected for ∼10 kb fragments according to the PacBio protocol. We used 4kb-5 kb cut off for the size selection, eliminating small fragments for sequencing. Even though the number of contigs is high for *S. sioyaensis* B-5408, the scaffold is intact, and bioinformatics was able to be completed with high confidence in the results.

### 3.2 *Streptomyces* species relatedness

Several phylogenetic studies on *Streptomyces* have been published in the past [54,55]. Here we added our species to the available 394 *Streptomyces* species at NCBI and constructed a phylogenetic tree based on full length 16S rRNA sequences (Fig. 1a). Due to the variability in sequencing technologies, only 94 species were reported with full-length 16S rRNA sequences in the NCBI RefSeq database. Our phylogenetic analysis shows the placement of our three sequenced species in three separate clades, as previously observed [[56], [57], [58]].

Next, we analyzed species relatedness based on whole genome nucleotide profiles using average nucleotide identity (ANI) [50]. This analysis resulted in a cluster pattern between the species (Fig. 1b) that indicate a unique clade distribution consistent with sub-family populations (Fig. 1a). *S. sioyaensis* demonstrates a high similarity to *S. auratus* at greater than 90 % (Fig. 1b,), as previously observed [55]. Our ANI analysis shows that *S. actuosus*, *A. acidiphila*, and *S. sioyaensis* have a less than 75 % nucleotide identity (Fig. 1b) which is typical for species within a genus. Together, these data indicate a high level of sequence divergence between the Actinomycetes reported here and previously published *Streptomyces* species.

### 3.3 Predicted protein family distribution

**Fig. 2:**
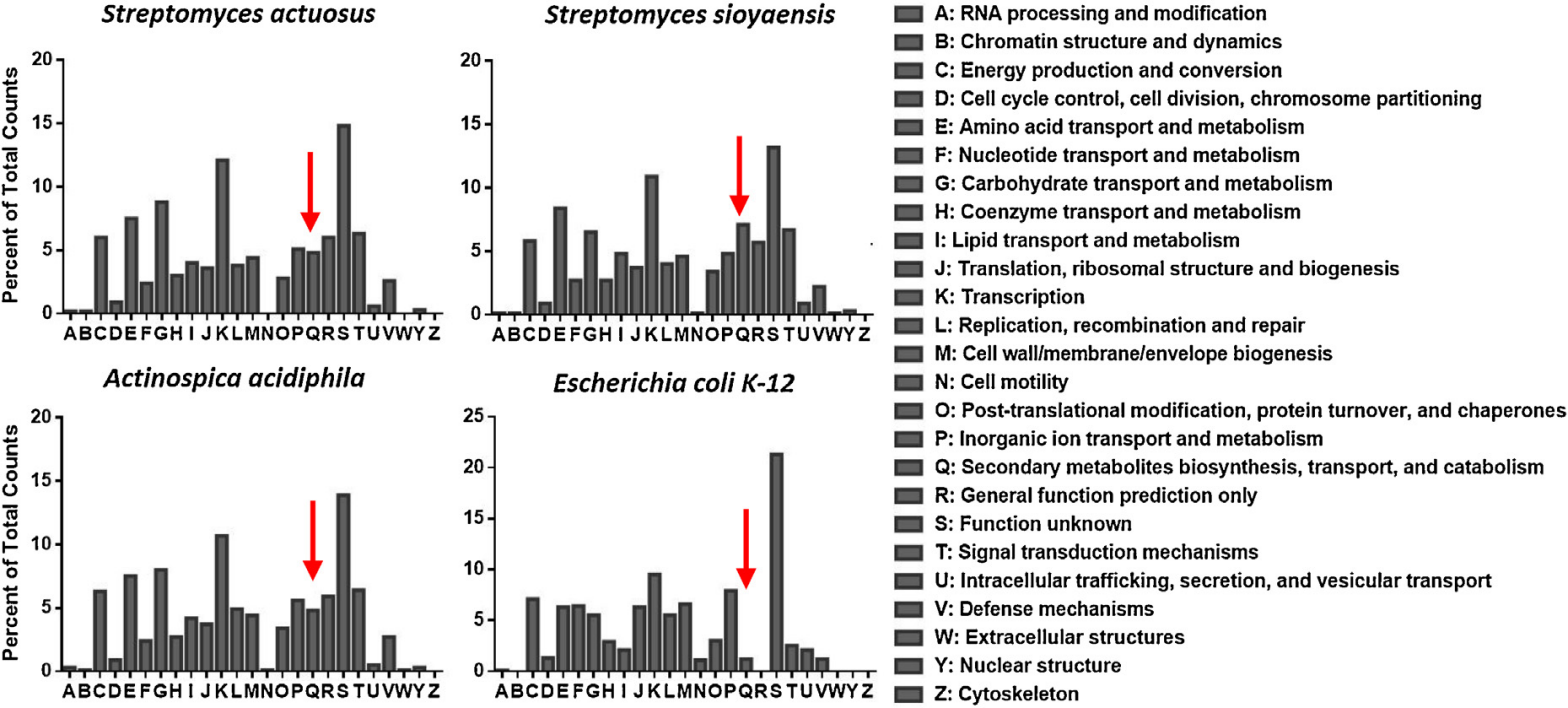
Predicted protein family distribution. Clusters of orthologous groups were identified in *S. actuosus* ISP-5337, *S. sioyaensis* B-5408, *A. acidiphila* B-2296, and *E. coli* K-12 MG-1655 by scanning the amino acid files using InterProScan [42]. The results from the InterProScan analysis were submitted to EggNOG [43] for annotation and classification of the protein families. The annotated protein families were plotted based on the percent of total predicted proteins per category. The number of proteins predicted to be involved in secondary metabolite biosynthesis (indicated by the red arrow) is 4 times as high in the Actinomycetes compared to the human pathogen *Escherichia coli* K-12 MG-1655.

Most proteins in each genome were predicted to have an ‘unknown’ function (Fig. 2, category S). Each sequenced species was predicted to maintain approximately 4.5–7% of their genome for proteins involved in secondary metabolite biosynthesis (category Q), while *Escherichia coli* K-12 sub-strain MG1655 only uses 1.2 % of its 5 Mb genome for secondary metabolism. Due to the high percentage of unknown proteins predicted per genome, the actual number of proteins involved in cellular processes may be underestimated.

Further analysis of the protein sequences by OrthoVenn2 [59] determined that the species form 5685 clusters, 2357 orthologous clusters (at least contains two species), and 3328 single-copy gene clusters (Fig. S1). All three species share 3507 orthologous clusters while 1887 clusters are shared between at least two of the genomes. A total of 229 gene clusters were specific to a single genome, with *S. sioyaensis* having the most clusters at 122, followed by *S. actuosus* with 92 and *A. acidiphila* with 77, respectively.

### 3.4 Predicted secondary metabolite biosynthetic gene clusters

We next predicted the presence of secondary metabolite biosynthetic gene clusters using antiSMASH [44]. AntiSMASH is a tool used to identify BGCs, including polyketide synthases, non-ribosomal peptide synthetases, terpenes synthases, clusters that produce RiPPs, and more. This analysis grouped the predicted proteins in Fig. 2 to functional clusters that encode machinery involved in the production of individual metabolites. antiSMASH analysis identified 17–32 BGCs across the three genomes with predicted functions ranging from compounds responsible for scavenging nutrients to antibiotics (Tables 2 and S1).

**Table 2:** Summary of the antiSMASH 5.0 results with a cut-off of >75 % similarity [44]. The percent similarity indicates the relatedness of the indicated BGC to the reference cluster on the MIBiG database [81]. The comprehensive list of antiSMASH results are listed in supplementary data Table S1.

Species	Total Clusters Predicted	BGC Product	% Similarity
*Streptomyces actuosus* ISP-5337	25	Nosiheptide	84
		Albaflavenone	100
		hatomarubigin A / hatomarubigin B / hatomarubigin C / hatomarubigin D	78
		Antimycin	100
		spore pigment	83
		Sarpeptin A / sarpeptin B	91
		Ectoine	100
		γ-butyrolactone	100
		hopene	92
		lagmysin	80
		informatipeptin	85
		desferrioxamin B / desferrioxamine E	83
*Streptomyces sioyaensis* B-5408	33	Siomycin	96
		Roseoflavin	100
		Desferrioxamine E	100
		Ectoine	100
		spore pigment	75
		Naringenin	100
		Citrulassin D	100
		Anantin C	75
		Phoslactomycin B	92
		iso-migrastatin / migrastatin / dorrigocin A / dorrigocin B / 13-epi-dorrigocin A	100
*Actinospica acidiphila* B-2296	17	Althiomycin	100
		Albaflavenone	100
		Antimycin	93
		Hopene	92
		Geosmin	100
		Sarpeptin A / sarpeptin B	91
		Desferrioxamin B / desferrioxamine E	83
		Ectoine	100
		spore pigment	83
		Alkylresorcinol	100

As expected, *S. sioyaensis* encodes for thiopeptide siomycin and *S. actuosus* encodes for the thiopeptide antibiotic nosiheptide [36]. *A. acidiphila* was predicted to encode known antibiotics althiomycin, albaflavenone, and antimycin (Table 2) along with two uncharacterized metabolites (Table S1). Interestingly, despite the ∼95 % similarity of the genomes, the secondary metabolite profile encoded by *A. acidiphila* B-2296 significantly differs from the secondary metabolite profile of its closest relative *A. acidiphila* B-24431 (Table S2); however, each strain is predicted to encode multiple antibiotics. This data supports the natural variation in secondary metabolomes observed across strains within a species and verifies the sub-family Actinospica as a reservoir for natural product antibiotics.

### 3.5 Metabolite analysis of culture extracts

The thiopeptide antibiotics produced by *S. sioyaensis* and *S. actuosus* are peptides that are heavily post-translationally modified [28]. The compounds are typically produced under stringent conditions and stored in the cell mycelia [25,51]. To extract metabolites, *S. actuosus*, *S. sioyaensis*, and *A. acidiphila* broth cultures were grown to a dense population and extracted with ethyl acetate (EtOAc). The production of siomycin and nosiheptide was determined by liquid chromatography coupled to mass spectrometry (LCMS). We also analyzed the extract of *A. acidiphila*, but in the absence of any previously characterized metabolites, this untargeted approach did not reveal any obvious hits (data not shown).

**Fig. 3:**
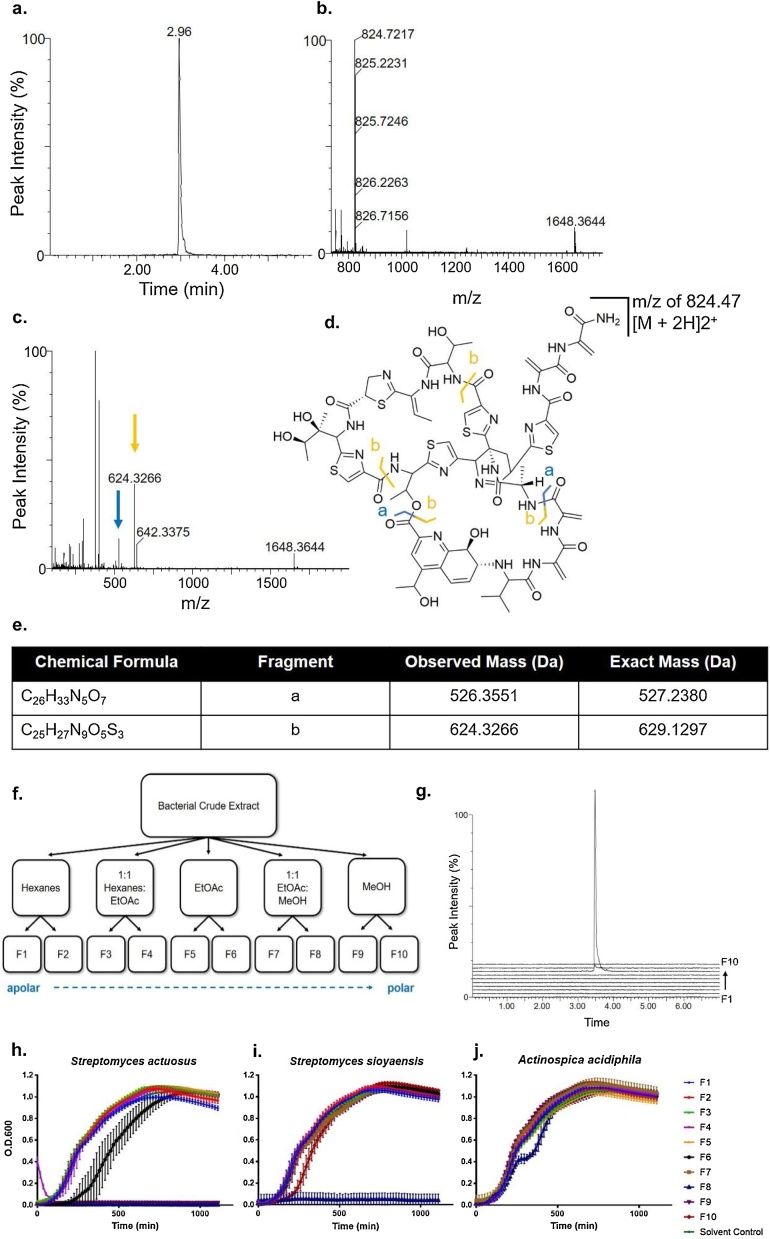
Mass spectrometry analysis of *Streptomyces sioyaensis* culture extract and antibacterial activity of *S. actuosus* ISP-5337, *S. sioyaensis* B-5408, and *A. acidiphila* B-2296 fractionated extracts challenged against *Staphylococcus aureus*. a) Siomycin extracted from the total ion count at a retention time of 2.96 min. b) Mass spectra under the 2.96 min chromatographic peak at 2.96 min shows the primary doubly charged isomers of siomycin at a *m/z* of 2 indicated by the 824.7217 Da peak and the parent compound and singly charged at 1648.3644 Da. c-e) MS^e^ data under the 2.96 min chromatographic peak at 2.96 min identified two fragment at *m/z* of 526.3551, blue cleavage pattern, and *m/z* of 624.3266, yellow cleavage pattern. f) Fractionation protocol used for elution of compounds by increasing polarity. g) Stacked extracted ion chromatogram extracted at 2.96 min, showing for siomycin that was identified in fraction 8 only. h-j) Growth curve analysis of *Staphylococcus aureus* survival by O.D.600 measurements at the indicated time interval (minutes) when challenged against fractions derived from *Streptomyces actuosus*, *Streptomyces sioyaensis*, and *Actinospica acidiphila*. Each assay was completed with three biological replicates (n = 3) and the standard deviation was calculated per timepoint.

Siomycin was observed in the culture extract at a retention time of 2.96 min (Fig. 3a) with a *m/z* of 824.7217 [M + 2 H]^2+^ (Fig. 3b). Analysis of MS^e^ data showed *m/z* hits of 526.3551 and 624.3266 Da, corresponding to the lower hemisphere of the molecule and the internal intersection of the macrocycles with the amino tail, respectively (Fig. 3c–e). To observe the breadth of antibiotics produced by *S. sioyaensis*, the crude extract was fractionated by flash chromatography over silica gel to separate the metabolites based on increasing polarity (Fig. 3f). Each fraction was analyzed by LCMS and siomycin was observed in fraction 8, which was eluted with a mixture of 50 % EtOAc and 50 % methanol, as expected based on the polarity of the natural product (Fig. 3g).

The *S. actuosus* culture was similarly extracted and processed for detection of the known metabolite nosiheptide. LCMS analysis showed the production of thiopeptide nosiheptide at a retention time of 2.76 min (Fig. S2a) with a *m/z* of 1222.1113 [M+H]^+^ (Fig. S2b). MS^e^ spectra analysis showed fragments with *m/z* values of 771.0480 and 1206.1369 Da, corresponding to the internal intersection of the macrocycles with the amino tail and a single fragment from the tail of nosiheptide, respectively (Fig. S2c–e). After fractionation, nosiheptide was also found in fractions 8–10 which eluted with a mixture of 50 % EtOAc: 50 % methanol and 100 % methanol from the column, matching its polarity (Fig. S2f–g).

### 3.6 Antibacterial activity assay against *Staphylococcus aureus*

The fractionated culture extracts were used to challenge the opportunistic pathogen *Staphylococcus aureus* ATCC 29213 (*S. aureus*) in a growth curve assay. When *S. aureus* was challenged with the fractions of *S. actuosus*, fraction 6 resulted in a significant growth delay while fractions 8–10 resulted in complete bactericidal activity (Fig. 3h). This data supports *S. actuosus* as a strong candidate for production of multiple antibiotic compounds against Gram positive bacteria, including, but not limited to, nosiheptide. *S. aureus* challenged with the fractions from *S. sioyaensis* displayed no growth in the presence of fraction 8 (Fig. 3i). By LCMS, fraction 8 was observed to contain siomycin (Fig. 3g), confirming antibacterial activity of siomycin against Gram positive bacteria. *A. acidiphila* culture extract did not show any antibiotic activity (Fig. 3j), despite bioinformatic analysis resulting in hits for antibiotics that target Gram positive bacteria in its genome (e.g. althiomycin [60,61]). In parallel, we tested the fractionated bacterial extracts against *Escherichia coli* DH5α and observed no antibacterial activity (Fig. S3). This result demonstrates the importance of the new approach to natural product discovery to identify antibiotics encoded in the genomes but not produced by these species under laboratory conditions. Together with the LCMS and bioassay analyses, each species was predicted and shown to produce antibiotics with potent activity against the common opportunistic pathogen *S. aureus*.

## 4 Discussion

Access to high quality whole genome sequences is a requirement for the new natural product discovery pipeline. Here, we sequenced three Actinomycetes that are known producers of thiopeptides, or in the family thereof, which not only revealed the BGCs for these thiopeptides but also a large amount of novel and uncharacterized BGCs (Tables 2 and S1). Considering the size of BGCs, it is important to have access to genomes with a small number of contigs to increase the likelihood that each BGC is contained within a single contig [20,62]. PacBio sequencing is well suited for this task since it produces long reads. PacBio is, indeed, quickly becoming the preferred method for sequencing *Streptomyces species* as shown by sequencing technology analysis of deposited genomes at NCBI (Fig. S4).

*S. actuosus* is known to produce nosiheptide and an avermipeptin analogue [24]. Nosiheptide is a thiopeptide, biosynthesized using both ribosomally synthesized and post-translationally modified peptide (RiPP) [63] and non-ribosomal peptide synthesis (NRPS) machineries [12,[64], [65], [66]]. Avermipeptin is a class III RiPP, characterized by cyclization of the linear peptide by LabKC cyclase that installs the thioether bridges [67]. This natural product was only discovered after whole genome sequencing of this species (Fig. S6) [24], showcasing the power of a genome mining approach. Both nosiheptide and the avermipeptin analogue are active against Gram positive bacteria like *S. aureus*. In line with this, we observe that polar fractions of *S. actuosus* inhibit growth of *S. aureus* (Fig. 3h). Besides nosiheptide and avermipeptin, *S. actuosus* is predicted to encode for albaflavenone, hatomarubigin, and antimycin at >75 % similarity (Tables 2 and S1). Together, this species encodes compounds with varying activities beneficial to human health [23,[67], [68], [69], [70], [71]].

*S. sioyaensis* is known to produce the thiopeptide siomycin. Besides encoding for siomycin, the previously reported genome (Fig. S7) of *S. sioyaensis* DSM 40032 shows 8 uncharacterized BGCs spread over 289 contigs [26]. In our data (split over 21 contigs), there were only 5 unknown BGCs predicted and the overall analysis resulted in fewer false positives (Table S1). Like nosiheptide, siomycin is active against Gram positive bacteria as we and others have shown (Fig. 3i) [[72], [73], [74]]. There is renewed interest in thiopeptide natural products like nosiheptide and siomycin due to their unique biosynthesis, mode of action, and underexploited utility as antibiotics [75]. The combination of both RiPP and NRPS biosynthetic machineries in one BGC is unique [12]. These thiopeptides target ribosomal subunits that are not targeted by any other antibiotics [36]; however, they are not being used in the clinic due to poor solubility and bioavailability [28,35,75]. Chemical modification or bioengineering approaches are being pursued to turn these natural products into next generation antibiotics.

A third member of the unique thiopeptides harboring both RiPP and NRPS character in their BGCs is thiostrepton. *S. azureus* and *S. cyaneus* CGMCC 4.1671 are known producers of thiostrepton [58,76,77]; however, whole genome sequencing of *S. cyaneus* B-2296^T^ revealed that the strain we received was misannotated as *S. cyaneus* and should be reclassified as *A. acidiphila* B-2296 (Fig. S5). *A. acidiphila* strains do not encode for any thiopeptides, but antiSMASH analysis shows the presence of 17 secondary metabolite BGCs, 9 of which encode metabolites with known antibiotic activity (Table S2). The whole genome relatedness of all reported *Actinospica* species against *A. acidiphila* B-2296 was analyzed by ANI (Fig. S5b). Interestingly, *A. acidiphila* B-24431 demonstrated the highest percentage of similarity to the *A. acidiphila* B-2296 species reported here with ∼95 % similarity; however, a relatively high percent similarity is observed across all Actinospica species. In parallel, a low percent similarity from *Streptomyces azureus* B-2655 and *Streptomyces cyaneus* CGMCC 4.1671 was observed. Coupled to the ANI analysis is Fig. 1b which demonstrated a unique cluster pattern surrounding *A. acidiphila* B-2296, this data suggests the presence of numerous *Streptomyces* species with a high percentage of similarity to the Actinospica sub-family.

The advent of affordable whole genome sequencing combined with a better understanding of biosynthesis, allows for computational mining of bacteria for natural product production [22]. The Actinomycetes species described in this work were isolated in the 1940s and 1950s and named based on growth characteristics. However, taxonomy and secondary metabolite profiles are often contradictory [78], emphasizing the need for a genomic approach to bacterial natural product discovery. Even *Streptomyces* species with identical 16S sequences show very different secondary metabolomes [79]. Together, our results demonstrate *S. sioyaensis*, *S. actuosus*, and *A. acidiphila* as reservoirs for multiple classes of natural products, some with potent antibiotic activity. With high quality genomes, our data facilitates a genomic approach to natural product discovery. Future studies will focus on development of the known thiopeptide antibiotics into clinically relevant compounds as well as expand the reservoir of potential new antibiotics not previously characterized from these organisms.

## 5 Conclusions

Whole genome sequencing and bioinformatic analyses of *Streptomyces sioyaensis*, *Streptomyces actuosus*, and *Actinospica acidiphila* revealed numerous secondary metabolite biosynthetic gene clusters. Liquid chromatography mass spectrometry coupled to antibiotic activity assays verified the production of potent thiopeptides siomycin and nosiheptide, produced by *S. sioyaensis* and *S. actuosus*, respectively. These results expand the field of natural product discovery and provide a genomic platform in which to bioengineer relevant new antibiotics to combat the antimicrobial resistance crisis.

## CRediT authorship contribution statement

**Haley M. Majer:** Investigation, Data curation, Formal analysis, Writing - original draft, Visualization, Software, Validation. **Rachel L. Ehrlich:** Data curation. **Azad Ahmed:** Investigation. **Joshua P. Earl:** Data curation. **Garth D. Ehrlich:** Funding acquisition. **Joris Beld:** Supervision, Funding acquisition, Conceptualization, Methodology, Software, Data curation, Writing - review & editing.

## Declaration of Competing Interest

The authors report no declarations of interest.
