# Supplementary material for: Whole genome sequencing of Streptomyces actuosus ISP-5337, Streptomyces sioyaensis B-5408, and Actinospica acidiphila B-2296 reveals secondary metabolomes with antibiotic potential
Source: Biotechnol Rep (Amst). 2021 Feb 9;29:e00596. doi: 10.1016/j.btre.2021.e00596 (PMC7893419; doi:10.1016/j.btre.2021.e00596)
Supplement: Supplementary file 1 [file mmc1.docx]

**Supplemental Information**

Whole Genome Sequencing of *Streptomyces actuosus* ISP-5337, *Streptomyces sioyaensis* B-5408, and *Streptomyces cyaneus* B-2296 Reveals Secondary Metabolomes with Antibiotic Potential

Haley M. Majer, Rachel L. Ehrlich, Azad Ahmed, Joshua P. Earl, Garth D. Ehrlich, and Joris Beld*

Department of Microbiology and Immunology, Drexel University College of Medicine, 245 N 15^th^ St, Philadelphia, PA 19102, USA

*Correspondence: jb3669@drexel.edu. Phone: +1 267 359 2355.

| **Organism** | **Cluster type** | **Most Similar to Known Cluster** | **Cluster Biosynthesis** | **Percent Similarity** | **Complete Sequence** |
| --- | --- | --- | --- | --- | --- |
| ***S. sioyaensis*** | hglE-KS,T1PKS | Roseoflavin | NRP + Polyketide | 100 | no |
|  | siderophore | Desferrioxamine E | Other | 100 | yes |
|  | ectoine | Ectoine | Other | 100 | yes |
|  | T1PKS | Thiolutin | NRP | 12 | yes |
|  | siderohpore | Unknown | Unknown | Unknown | yes |
|  | NRPS, other | Deimino-antipain | NRP | 66 | no |
|  | terpene | Isorenieratene | Terpene | 71 | yes |
|  | melanin | A-500359 A / A-500359 B | NRP | 5 | no |
|  | PKS-like,T1PKS,NRPS-like,oligosaccharide,butyrolactone | Cyphomycin | Polyketide | 61 | yes |
|  | T2PKS | spore pigment | Polyketide | 75 | no |
|  | bacteriocin | Unknown | Unknown | Unknown | yes |
|  | T3PKS | Naringenin | Terpene | 100 | yes |
|  | bacteriocin | Unknown | Unknown | Unknown | yes |
|  | NRPS | a201a | Other: Nucleoside | 8 | no |
|  | T1PKS | Sceliphrolactam | Polyketide | 32 | no |
|  | butyrolactone | Blasticidin S | Other | 7 | yes |
|  | T3PKS | Daptomycin | NRP | 7 | yes |
|  | T1PKS, hglE-KS | Herboxidiene | Polyketide | 4 | yes |
|  | terpene | Hopene | Terpene | 69 | yes |
|  | bacteriocin | Unknown | Unknown | Unknown | yes |
|  | butyrolactone | Unknown | Unknown | Unknown | yes |
|  | ladderane, lassopeptide | Citrulassin D | RiPP | 100 | yes |
|  | siderophore | Ficellomycin | NRP | 3 | yes |
|  | linaridin | Pentostatine/vidarabine | Other | 6 | yes |
|  | linaridin | Legonaridin | RiPP | 66 | yes |
|  | Lassopeptide, terpene | Anantin C | RiPP | 75 | yes |
|  | Terpene, thiopeptide, LAP | Siomycin | RiPP: Thiopeptide | 96 | yes |
|  | butyrolactone,phenazine,PKS-like | Esmeraldin | Polyketide + Other:Aminocoumarin | 64 | yes |
|  | NRPS | Lipstatin | NRP | 35 | no |
|  | T1PKS | Phoslactomycin B | Polyketide | 92 | no |
|  | transAT-PKS | iso-migrastatin / migrastatin / dorrigocin A / dorrigocin B / 13-epi-dorrigocin A | Polyketide:Modular type I + Polyketide:Trans-AT type I | 100 | no |
|  | butyrolactone | Coelimycin P1 | Polyketide:Modular type I | 12 | yes |
|  | T1PKS | Sceliphrolactam | Polyketide | 56 | no |
| 1. ***acidiphila*** | LAP,T2PKS,PKS-like,NRPS-like,betalactone | LL-D49194α1 (LLD) | Polyketide | 49 | no |
|  | bacteriocin | Informatipeptin | RiPP: Lanthipeptide | 57 | yes |
|  | teprene | Hopene | Terpene | 92 | yes |
|  | siderophore | Grincamycin | Polyketide:Type II + Saccharide:Hybrid/tailoring | 8 | yes |
|  | NRPS, T1PKS | Althiomycin | NRP + Polyketide:Modular type I | 100 | yes |
|  | terpene | Geosmin | Terpene | 100 | yes |
|  | bacteriocin | Unknown | Unknown | Unknown | yes |
|  | NRPS | Sarpeptin A / sarpeptin B | NRP | 91 | yes |
|  | terpene | Albaflavenone | Terpene | 100 | yes |
|  | siderophore | Desferrioxamin B / desferrioxamine E | Other | 83 | yes |
|  | NRPS | SCO-2138 | RiPP | 64 | yes |
|  | ectoine | Ectoine | Other | 100 | yes |
|  | T2PKS | spore pigment | Polyketide | 83 | yes |
|  | terpene | Carotenoid | Terpene | 54 | yes |
|  | T3PKS | Alkylresorcinol | Polyketide | 100 | yes |
|  | lanthipeptide | Unknown | Unknown | Unknown | yes |
|  | NRPS, T1PKS | Antimycin | NRP + Polyketide | 93 | no |
| ***S. actuosus*** | ectoine | Ectoine | Other | 100 | yes |
|  | arylpolyene | Tetronasin | Polyketide | 5 | yes |
|  | T2PKS | spore pigment | Polyketide | 83 | yes |
|  | PKS-like, butyrolactone | Simocyclinone D8 | Saccharide + Polyketide:Modular type I + Polyketide:Type II + Other:Aminocoumarin | 8 | yes |
|  | furan | Methylenomycin A | Other | 9 | yes |
|  | Thiopeptide, LAP | nosiheptide | RiPP: Thiopeptide | 84 | yes |
|  | NRPS | Sarpeptin A / sarpeptin B | NRP | 91 | yes |
|  | terpene | albaflavenone | Terpene | 100 | yes |
|  | Siderophore | Unknown | Unknown | Unknown | yes |
|  | Bacteriocin | Unknown | Unknown | Unknown | yes |
|  | Butyrolactone, terpene | γ-butyrolactone | Other | 100 | yes |
|  | T1PKS, siderophore | paulomycin | Other | 11 | yes |
|  | bacteriocin | α-lipomycin | Saccharide + Polyketide:Modular type I + Polyketide:Type II + Other:Aminocoumarin | 9 | yes |
|  | Other | A-503083 A / A-503083 B / A-503083 E / A-503083 F | NRP | 7 | yes |
|  | terpene | hopene | Terpene | 92 | yes |
|  | NRPS | leinamycin | NRP + Polyketide:Modular type I + Polyketide:Trans-AT type I | 2 | yes |
|  | lassopeptide | lagmysin | RiPP | 80 | yes |
|  | Lanthipeptide, bacteriocin | informatipeptin | RiPP: Lanthipeptide | 85 | yes |
|  | siderophore | desferrioxamin B / desferrioxamine E | Other | 83 | yes |
|  | melanin, T1PKS, butyrolactone | 4-hexadecanoyl-3-hydroxy-2-(hydroxymethyl)-2H-furan-5-one | Polyketide | 45 | yes |
|  | T2PKS, butyrolactone, indole | hatomarubigin A / hatomarubigin B / hatomarubigin C / hatomarubigin D | Polyketide | 78 | yes |
|  | terpene | carotenoid | Terpene | 63 | yes |
|  | melanin | melanin | Other | 71 | yes |
|  | b-lactam | A83543A | Polyketide | 8 | yes |
|  | NRPS, T1PKS, T3PKS | antimycin | NRP + Polyketide | 100 | yes |

Supplementary Table 1: Total secondary metabolite biosynthetic gene clusters predicted by antiSMASH 5.0 include Organism, Cluster type, Most Similar to Known Cluster, Cluster Biosynthesis, Percent Similarity, and Complete Sequence for organisms *S. sioyaensis*, *A. acidiphila*, and *S. actuosus* [1]. Cluster type specifies the class of secondary metabolite biosynthetic gene cluster (smBGC). Most similar to known cluster denotes the metabolite that is produced by the most similar cluster. Cluster biosynthesis outlines the mechanism of metabolite production. Percent similarity indicates the similarity of the predicted clusters to the reference cluster sequence in the antiSMASH database. Complete sequence, indicated by yes or no, signifies whether the cluster was completely sequenced. “*Yes”* clusters were observed to be located centrally within a contig with completely sequenced flanking regions. “*No”* clusters were observed to be located on the edges of the contigs and the confidence in completely sequenced flanking regions is low.


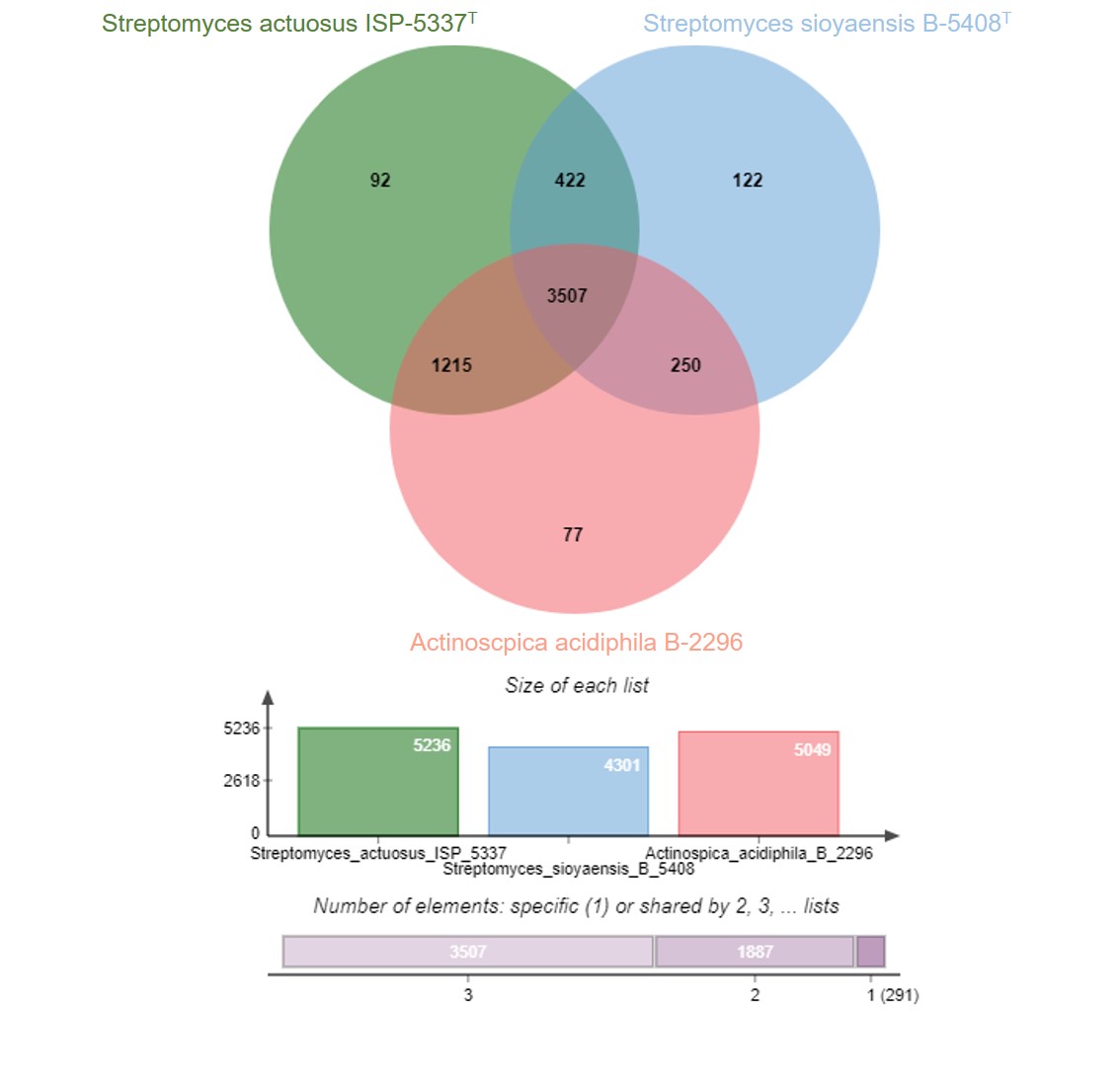


Supplementary Figure 1: Distribution of shared and unique gene clusters observed between *Streptomyces actuosus* ISP-5337T, *Streptomyces sioyaensis* B-5408T, and *Actinospica acidiphila* B-2296 by OrthoVenn2 analysis [2].


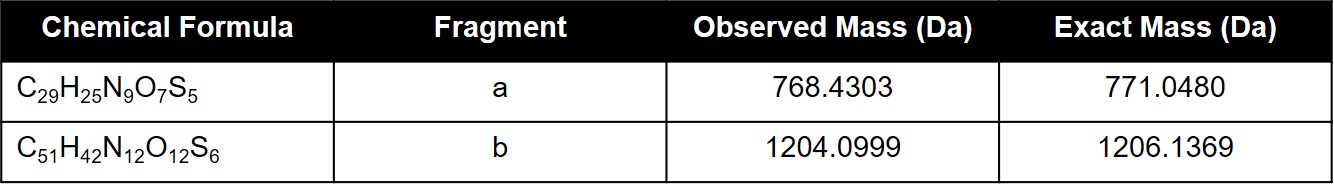


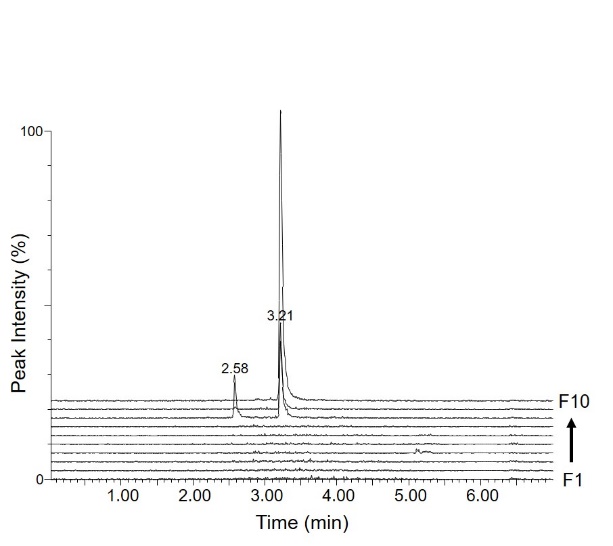

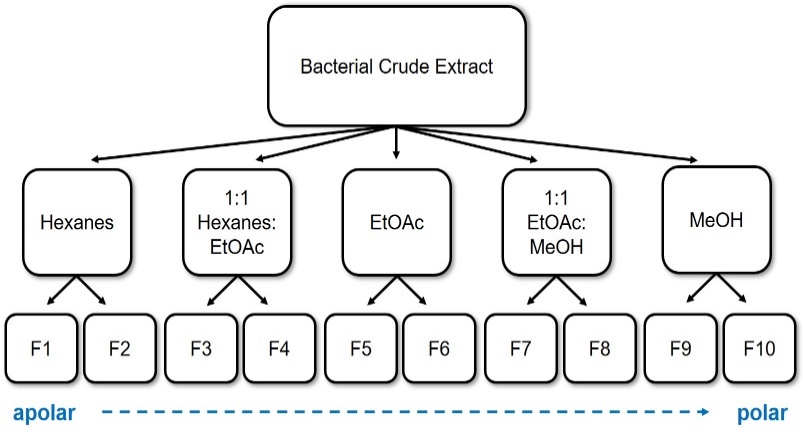

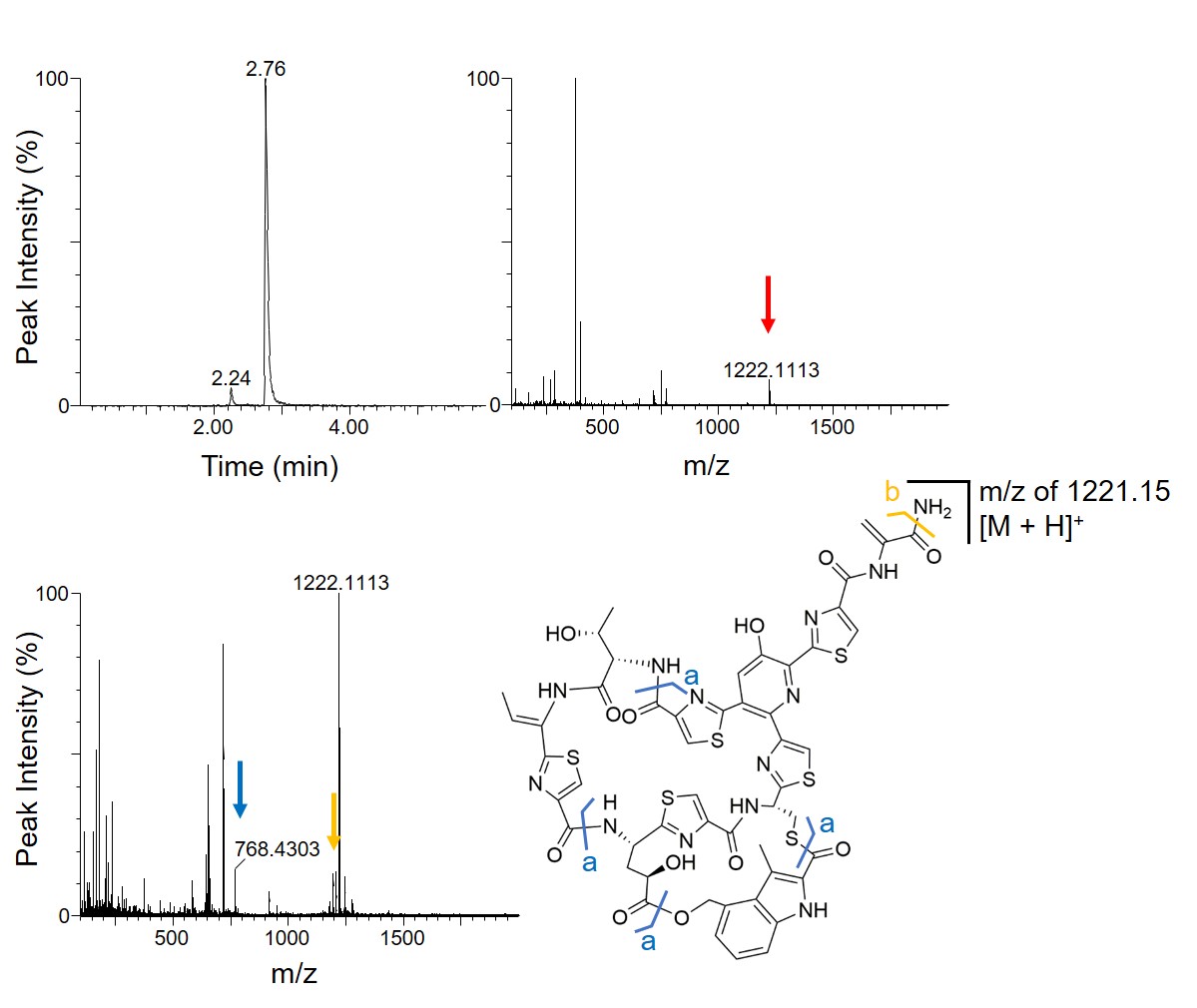


**g.**

**f.**

**e.**

**d.**

**c.**

**b.**

**a.**

Supplemental Figure 2: Mass spectrometry analysis of *Streptomyces actuosus* culture extract. a) Nosiheptide extracted from the total ion count at a retention time of 2.76 minutes. b) Spectra under the 2.76 minute chromatographic peak shows nosiheptide at a m/z of 1222.1113 Da. c-e) MS^e^ data under the 2.76 minute chromatographic peak identified two fragment at m/z of 768.4303, blue cleavage pattern, and m/z of 1204.0999, yellow cleavage pattern. f) Fractionation protocol used for elution of compounds by increasing polarity. g) Stacked extracted ion chromatogram for nosiheptide. A shift in retention time was observed due to a change in UPLC pre-column setup (from a 0.2 µm filter to a Vanguard guard column). Nosiheptide was observed in the crude extract and fractions 8 through 10.


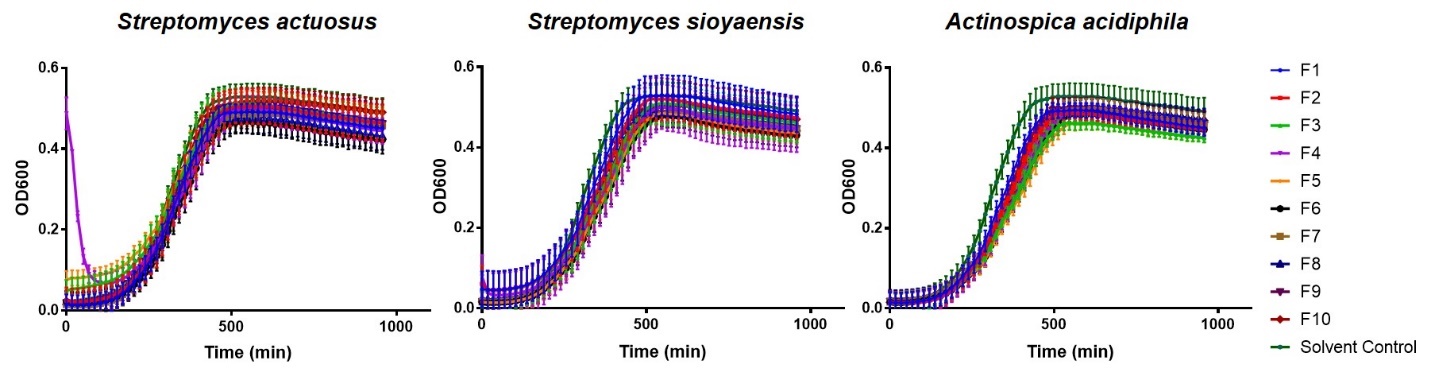
Supplemental Figure 3: Growth curve analysis of *Escherichia coli* survival by O.D.600 measurements at the indicated time interval (minutes) when challenged against fractions derived from *Streptomyces actuosus*, *Streptomyces sioyaensis*, and *Actinospica acidiphila*. Each assay was completed with three biological replicates (n=3) and the standard deviation was calculated per timepoint.


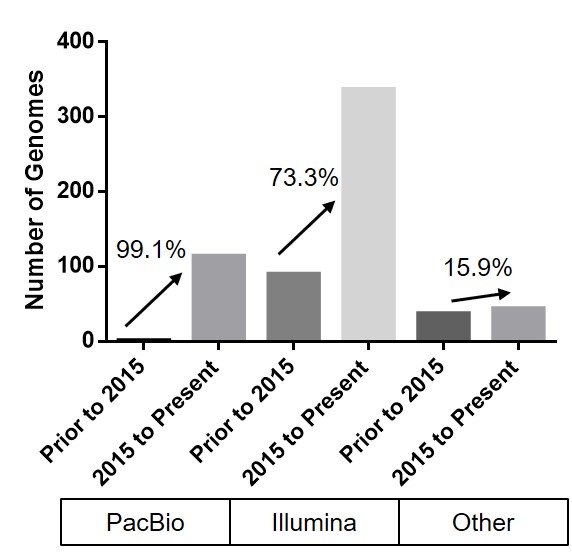


Supplementary Figure 4: Whole genome sequences of Streptomyces uploaded to NCBI categorized by sequencing technology and year.

| **Organism** | **Cluster type** | **Most Similar to Known BGC of** | **Percent Similarity** |
| --- | --- | --- | --- |
| ***Actinospica acidiphila* B-24431** | terpene | albaflavenone | 100 |
|  | siderophore | grincamycin | 8 |
|  | terpene | geosmin | 100 |
|  | bacteriocin |  |  |
|  | lanthipeptide |  |  |
|  | T3PKS | alkylresorcinol | 100 |
|  | terpene | carotenoid | 54 |
|  | siderophore | desferrioxamin B/desferrioxamine E | 83 |
|  | bacteriocin | informatipeptin | 57 |
|  | ectoine | ectoine | 100 |
|  | NRPS | SCO-2138 | 64 |
|  | T2PKS | spore pigment | 83 |
|  | T2PKS, PKS-like, betalactone | LL-D49194alpha1 | 45 |
|  | NRPS, T1PKS | antimycin | 100 |
|  | NRPS | auroramycin | 11 |
|  | NRPS-like, PKS-like | LL-D49194alpha1 | 3 |
|  | NRPS | sarpeptin A/sarpeptin B | 41 |
|  | terpene | hopene | 61 |
|  | NRPS | marformycinA/B/C/D/E/F | 12 |
|  | terpene | albaflavenone | 100 |

Supplementary Table 2: Total secondary metabolite biosynthetic gene clusters predicted by antiSMASH 5.0 [1] include cluster type, known cluster, and percent similarity for *Actinospica acidiphila* B-24431.

**a.**


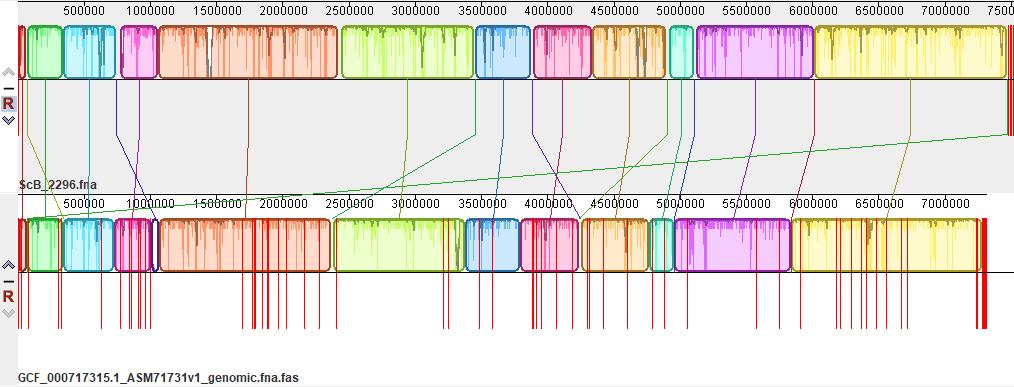


**b.**


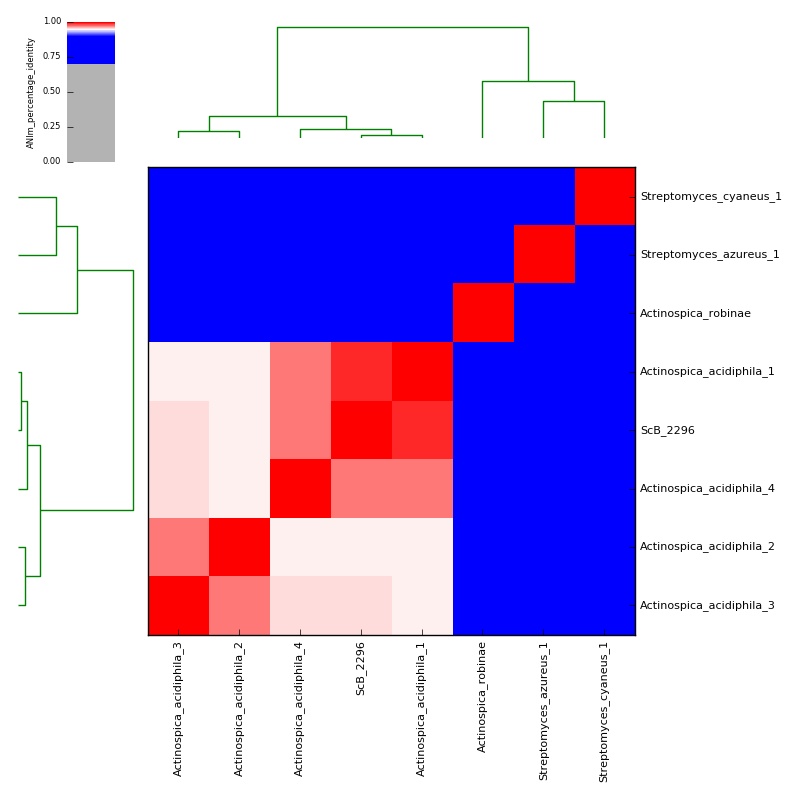


Supplementary Figure 5: a) *A. acidiphila* B-2296 (ScB_2296) was aligned to *A. acidiphila* B-24431 (GCF_000717315.1_ASM71731v1_genomic.fna) using progressiveMAUVE version 2.4.0 [3]. b) The average nucleotide identity (ANI) of *Streptomyces cyaneus* CGMCC 4.1671 (Streptomyces_cyaneus_1), *Streptomyces azureus* B-2655 (Streptomyces_azureus_1), *Actinospica robinae* DSM 44927 (Actinospica_robinae), *Actinospica acidiphila* B-24431 (Actinospica_acidiphila_1), *Actinospica acidiphila* B-2296 (ScB_2296), *Actinospica acidiphila* SID13773 (Actinospica_acidiphila_2), *Actinospica acidiphila* SID14190 (Actinospica_acidiphila_3), and *Actinospica acidiphila* SID8189 (Actinospica_acidiphila_4) was analyzed using PYANI 0.2.10 method MUMmer [4].


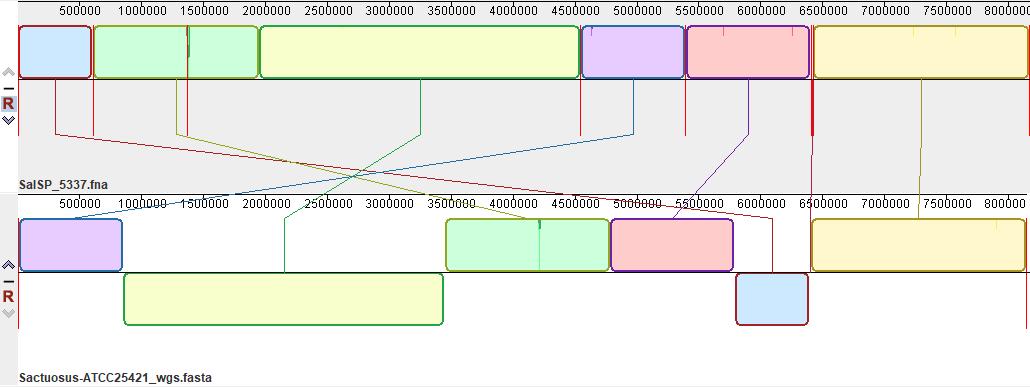


Supplementary Figure 6: Alignment of the published *S. actuosus* ATCC 25421 [5] with *S. actuosus* ISP-5337 (SaISP_5337) was completed using progressiveMAUVE version 2.4.0 [3].


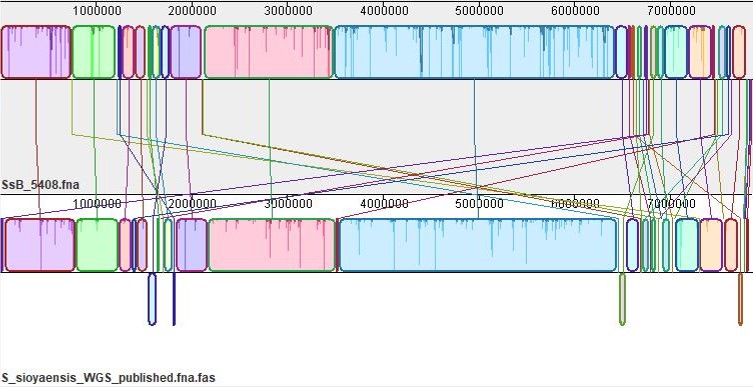


Supplemental Figure 7: *Streptomyces sioyaensis* DSM 40032 whole genome sequence was recently published using Illumina HiSeq [6]. Genome analysis classified the strain as *Streptomyces sioyaensis* DSM 40032 which is also designated as *S. sioyaensis* B-5408, the laboratory strain presented in this paper. Alignment of the published *S. sioyaensis* DSM 40032 (S_sioyaensis_WGS_published.fna) with *S. sioyaensis* B-5408 (SsB_5408) was completed using progressiveMAUVE version 2.4.0 [3].

1. Blin, K., et al., *antiSMASH 5.0: updates to the secondary metabolite genome mining pipeline.* Nucleic Acids Res, 2019. **47**(W1): p. W81-W87.

2. Xu, L., et al., *OrthoVenn2: a web server for whole-genome comparison and annotation of orthologous clusters across multiple species.* Nucleic Acids Res, 2019. **47**(W1): p. W52-W58.

3. Darling, A.E., B. Mau, and N.T. Perna, *progressiveMauve: multiple genome alignment with gene gain, loss and rearrangement.* PLoS One, 2010. **5**(6): p. e11147.

4. Pritchard, L., et al., *Genomics and taxonomy in diagnostics for food security: soft-rotting enterobacterial plant pathogens.* Anal. Methods, 2016. **8**(1): p. 12-24.

5. Liu, W., F. Sun, and Y. Hu, *Genome Mining-Mediated Discovery of a New Avermipeptin Analogue in Streptomyces actuosus ATCC 25421.* ChemistryOpen, 2018. **7**(7): p. 558-561.

6. Nakaew, N., et al., *Bioactivities and genome insights of a thermotolerant antibiotics-producing Streptomyces sp. TM32 reveal its potentials for novel drug discovery.* Microbiologyopen, 2019. **8**(11): p. e842.
